# Differential Analysis of Proteins Involved in Ester Metabolism in two *Saccharomyces cerevisiae* Strains during the Second Fermentation in Sparkling Wine Elaboration

**DOI:** 10.3390/microorganisms8030403

**Published:** 2020-03-13

**Authors:** Maria del Carmen González-Jiménez, Jaime Moreno-García, Teresa García-Martínez, Juan José Moreno, Anna Puig-Pujol, Fina Capdevilla, Juan Carlos Mauricio

**Affiliations:** 1 Department of Microbiology, University of Cordoba, 14014 Cordoba, Spain; b02gojim@uco.es (M.d.C.G.-J.); b62mogaj@uco.es (J.M.-G.); mi1gamaj@uco.es (J.C.M.); 2 Department of Agricultural Chemistry, University of Cordoba, 14014 Cordoba, Spain; qe1movij@uco.es; 3 Department of Enological Research, Institute of Agrifood Research and Technology-Catalan Institute of Vine and wine (IRTA-INCAVI), 08720 Barcelona, Spain; anna.puig@irta.cat (A.P.-P.); fcapdevila@gencat.cat (F.C.)

**Keywords:** *Saccharomyces cerevisiae*, sparkling wine, second fermentation, esters, proteins, CO_2_ overpressure, flor yeast1

## Abstract

The aromatic metabolites derived from yeast metabolism determine the characteristics of aroma and taste in wines, so they are considered of great industrial interest. Volatile esters represent the most important group and therefore, their presence is extremely important for the flavor profile of the wine. In this work, we use and compare two *Saccharomyces cerevisiae* yeast strains: P29, typical of sparkling wines resulting of second fermentation in a closed bottle; G1, a flor yeast responsible for the biological aging of Sherry wines. We aimed to analyze and compare the effect of endogenous CO_2_ overpressure on esters metabolism with the proteins related in these yeast strains, to understand the yeast fermentation process in sparkling wines. For this purpose, protein identification was carried out using the OFFGEL fractionator and the LTQ Orbitrap, following the detection and quantification of esters with gas chromatograph coupled to flame ionization detector (GC-FID) and stir-bar sorptive extraction, followed by thermal desorption and gas chromatography-mass spectrometry (SBSE-TD-GC-MS). Six acetate esters, fourteen ethyl esters, and five proteins involved in esters metabolism were identified. Moreover, significant correlations were established between esters and proteins. Both strains showed similar behavior. According to these results, the use of this flor yeast may be proposed for the sparkling wine production and enhance the diversity and the typicity of sparkling wine yeasts.

## 1. Introduction

Sparkling wine is an ancient beverage appreciated for its unique and pleasant taste. This is attributed to aroma compounds that are produced during fermentation, highlighting the higher alcohols and esters. Esters are the primary source of fruity aromas and extremely important for the flavor profile of the wine. The main pathway that leads to the formation of aroma compounds that contribute to the wine organoleptic properties are the Ehrlich pathway for higher alcohols or the enzymes responsible for the formation of esters. These biochemical pathways were studied mainly in yeast species [1] that include more than 2000 yeast species; some of them have different and potentially interesting yeast species for the food and beverage industries [2].

Over the years, attempts have been performed to determine the biochemical mechanisms of formation of volatile sparkling wine compounds during the first and second fermentation [3,4,5]. The formation of esters during fermentation is a dynamic process [6]. Particularly, the concentration of esters produced depend on the yeast strain [7,8,9,10,11,12], fermentation temperature [13,14], the material insoluble in the must [11,15], winemaking methods [16,17,18,19], pH [20], the amount of SO_2_ [11,21], the amino acids present in the must [11,22,23], the glucose and dissolved oxygen [24], malolactic fermentation [25,26], and the presence of lees in the wine [27,28]. The maximum concentration of esters is obtained when yeasts reach the stationary growth phase [6]. Esters can be divided into two main groups. The first group is constituted by acetate esters, in which acetate is the acid group and ethanol or an alcohol complex derived from the metabolism of the amino acids, are the alcohol group. Some compounds of this group are ethyl acetate (solvent-similar aroma), isoamyl acetate (banana aroma), and phenyl ethyl acetate (roses or honey aroma) [29]. The second group of esters is formed by the ethyl esters. In this one, the alcohol group is ethanol and the acid group is a medium-chain fatty acid. Ethyl hexanoate (anise seed), ethyl octanoate (acid apple aroma), ethyl decanoate (floral aroma), and ethyl lactate (milk, soapy, buttery, fruity) are included in this group [27]. Aroma thresholds (mg/L) are described in Sumby et al. (2010) [30].

The enzymatic accumulation of esters is the result of the balance of synthesis and hydrolysis enzymatic reactions. These enzymatic activities are very low during the fermentation of grape must [8,24,31]. *S. cerevisiae* genome encodes five known genes (*ATF1*, *ATF2*, *EHT1*, *EEB1*, and *IAH1*) that are involved in ester synthesis and hydrolysis. Atf1p, Atf2p, Eht1p, and Eeb1p are proteins implied in ester synthesis and Iah1p is, primarily, involved in acetate esters hydrolysis [30]. The role of Atf1p and Atf2p in acetate ester synthesis is clear, however, the role of Eth1p and Eeb1p in ethyl ester synthesis needs to be more accurately defined. The synthesis of acetate esters in *S. cerevisiae* occurs during the fermentation and it is highly associated with lipid metabolism and yeast growth. These compounds are synthesized in the cytoplasm as result of reactions catalyzed by acyl transferase enzymes, in which Acyl-CoA is required as a co-substrate. Most of the required Acyl-CoA is generated by oxidative decarboxylation of pyruvate, giving rise to Acetyl-CoA, while the rest of Acyl-CoA is formed by the acylation between fatty acids and free coenzyme A (CoA). In the absence of oxygen, the reaction between Acetyl-CoA and alcohol (ethanol or higher alcohols) allows the formation of acetate esters, while the combination between the long chains of Acyl-CoA and ethanol produce ethyl esters. Once synthesized within the cell, by their lipophilic nature, the esters can diffuse through the cell membrane to be released into the fermentation medium [29].

The final concentration of acetate esters is also influenced by the presence of esterases, a group of hydrolase enzymes that catalyze the breakdown of esters or prevent their formation [8,29]. Particularly, the Iah1p enzyme plays an important role in the hydrolysis of isoamyl acetate, ethyl acetate, isobutyl acetate, and phenylethyl acetate. As it was previously reported, esters are extremely important in the taste of wines. It is known that these metabolites are in very low concentrations, close to their threshold values [30]. The fact that most esters are present in such concentrations implies that small changes in the concentration could have a relevant effect on the taste of the wine.

This study is based on previous works by our research group [32,33] aimed to study the general aroma compound profile of P29 and G1. Specifically, we focused on the esters because of their positive impact on the organoleptic properties and provide, for the first time, the proteins involved in their metabolism and a metabolome–proteome relation during the second fermentation in the production of sparkling wine (cava wine). The use of unconventional yeast strains to produce new or improved fermented beverages, such as sparkling wines, is an interesting approach according to several authors [12,34,35,36]. The use of a flor yeast for the production of sparkling wine would be interesting due to its high tolerance to ethanol or its adhesive properties that facilitate its better recovery in the “degüelle” phase, making the flor yeast ideal for a second fermentation in the bottle. This would mean a reduction in production costs and manufacturing time, an increase in the biodiversity of yeast strains in the production of sparkling wine and typicity elaborated wines with an own flor yeast strain from DO Montilla-Moriles (south of Spain). Furthermore, this study helps to understand how endogenous CO_2_ overpressure can affect the sensory characteristics of sparkling wines during the second fermentation.

## 2. Materials and Methods

### 2.1. Microorganisms and Conditions Studied

Two strains of *S. cerevisiae* were used: G1 strain (ATCC: MYA-2451) is a wine flor yeast isolated from a flor velum in a barrel under biological aging of “Fino” Sherry wine of the designation of origin (DO) Montilla-Moriles in Cordoba (Spain). The other one is the P29 strain (CECT11770), typical of sparkling wines resulting of second fermentation in a closed bottle. It was isolated from Penedès designation of origin (DO) Barcelona (Spain).

For the inoculum, a total of 1 x 10^6^ yeast cells/mL, which were previously grown in medium YPD (1% yeast extract, 2% peptone, 2% dextrose) at 21 °C for 48 h, were incubated for 5 days at 21 °C using gentle agitation of 100 rpm for their growth in a pasteurized must of Macabeo grape variety (174.9 g/L of sugar, 18.5 °Bx, 3.6 g/L of total acidity, and 3.43 pH). When ethanol content of 10.39 % (v/v) was reached, the yeast cells were inoculated into bottles for the run along with a standardized commercial base wine, 21 g/L sucrose, and 1.5 x 10^6^ cells/mL. This base wine is composed of Macabeo:Chardonnay (6:4), 10.21 % (v/v) of ethanol, 0.3 g/L of sugar, pH 3.29, 5.4 g/L of total acidity, and 0.21 g/L of volatile acidity. The second fermentation was carried out in a thermostatic chamber at 14 °C in bottles with a volume of 750 mL. In order to study the effect of CO_2_ released by yeasts during this fermentative process, the bottles were divided into groups for the study of endogenous CO_2_ overpressure. Three bottles were used for proteomic and metabolomic analyses at each sampling time: BW, MFP (+), EFP (+), MFP (−), and EFP (−), with a total of 15 bottles for each strain. Therefore, a total of 30 bottles were used.

For the condition with CO_2_ pressure, the bottles were hermetically sealed with a plug and with a metal crown capsule, hereinafter referred to as a pressure condition, P (+); the bottles belonging to the condition without pressure were closed with a perforated shutter, constituting the condition without CO_2_ pressure, P (−). During the process, the following samples were taken: (a) at the beginning of the inoculation in the base wine, BW; (b) in the middle of the second fermentation, MF (3 atm pressure); (c) at the end of the second fermentation, EF (6.5 atm). At the same time, samples of the condition without CO_2_ pressure were taken, obtaining a content of sugar consumed and ethanol produced similar for both conditions: sugar consumption (MF: 9.07 ± 0.26 g/L and EF: 0.3 ± 0.0 g/L) and ethanol content (MF: 10.74 ± 0.03% v/v and EF: 11.56 ± 0.04% v/v).

Cell viability was determined by spreading 100 µL volumes of diluted suspension on YPD (yeast extract, peptone, dextrose) agar plates and counting colonies after 48 h at 28 °C.

### 2.2. Analysis of Volatile Metabolites

The detection of volatile compounds in wine is based on the different physico-chemical properties such as volatility or solubility in different organic phases. Majority compounds do not need to be pre-concentrated beforehand, since they have a high enough concentration to be detected. Ethyl acetate and ethyl lactate were detected using the direct injection method in an Agilent 6890 Series II gas chromatograph equipped with a fused silica capillary column CP-WAX 57 CB (60 m long, 0.25 mm internal diameter, and 0.4 μm film thickness) attached to a FID detector. The temperature program was as it follows: 50 °C for 15 min and then raised to 190 °C at 4 °C/min for 35 min. The flow rate of the carrier gas (helium) was held at 0.7 mL/min for 16 min and then raised at 0.2 to 1.1 mL/min for 52 min. The flame ionization detector temperature was 300 °C, and the hydrogen and air flow rates were 40 and 400 mL/min, respectively. A post run purge program at 200 °C for 35 min and a helium flow rate of 1.3 mL/min were used after the chromatographic peaks of interest were eluted. A solution of 1 g/L of 4-methyl-2-pentanol in pure ethanol was used as internal standard. These chromatographic conditions are described in Peinado et al. (2004) [37].

The other group of volatile compounds analyzed was those found in low concentrations, less than 10 mg/L, that need to be extracted and concentrated in order to detect them. These volatile compounds are called minority compounds and were identified using the SBSE (stir bar sorptive extraction), followed by thermal desorption and gas chromatography coupled to a mass spectrometer (SBSE-TD-GC-MS). The SBSE-TDU-GC-MS analytical platform consisted of an Agilent-7890ª chromatograph, a MSD 5975 mass detector (Agilent Technologies), and the Gerstel thermal desorption unit (TDU) coupled to a CIS-4 injection system. Agilent and an HP-5 capillary column 30 m long, 0.25 mm internal diameter, and 0.25 μm film thickness. This technique uses a magnetic stirring bar, called Twister (0.5 mm thick coated and 10 mm long) packed with polydimethylsiloxane (PDMS). A 10 mL vial was filled with 0.5 mL of sample and 0.1 mL of an internal standard solution (0.4464 mg/L ethyl nonanoate in pure ethanol plus a solution of 12% (v/v) ethanol adjusted to pH 3.5 with 2.6 g/L tartaric acid and 2.2 g/L potassium bitartrate). The final volume was 10 mL. Compounds were thermally desorbed at an initial temperature of 35 °C for 0.1 s, using a 120 °C/min ramp to 280 °C for 10 min and a helium stream at 16 mL/min in the splitless mode into a cooled injection system (CIS-4) from Agilent Technologies. The 7890A GC instrument was equipped with an HP5MS fused silica capillary column (30 m × 0.25 mm) from Agilent Technologies (Wilmington, DE, USA). The oven initial temperature was set at 50 °C for 2 min and then increased with 4 °C/min to a final temperature of 190 °C that was maintained for 10 min. The MSD was used at 70 eV in the electron impact mode (EI), using the mass range from 35 to 550 Da at 150 °C. More detailed information is described in other publications [38,39].

### 2.3. Proteomic Analysis

#### Extraction, Identification, and Quantification of Proteins

The cells were collected from each bottle (data shown in Table 1) by centrifugation at 4500× *g* for 10 min by a centrifuge (Rotina-38), washing the sediment twice with sterile distilled cold water. Afterwards, a total of 2 x 10^9^ cells of each condition counted with Beckman Coulter Z2 Particle Counter were used for protein extraction. This amount of yeast cells was broken by a mechanical technique in Vibrogen Cell Mill V6 (Edmund Bühler) using 500 µm diameter glass balls. Once the cells were broken, the protein pull was extracted. For this purpose, extraction buffer (100 mM Tris-HCl pH 8, 0.1 mM EDTA, 2 mM DTT, and 1 mM PMSF) and a protease inhibitor cocktail were used.

Later, the protein concentration was estimated by the Bradford test (1976) [40] to subsequently proceed to protein analysis. To perform this analysis, 500 µg of total protein from each condition and replica was loaded into the tray of the well of the OFFGEL 3100 fractionator from Agilent Technologies. Previously the protein samples were solubilized in Protein OFFGEL fractionation buffer containing urea, thiourea, DTT, glycerol, and buffer with ampholytes. The aliquots were distributed in the OFFGEL 3100 fractionator from Agilent Technologies, in a tray with wells. The separation limits of the OG12PR00 program that were used were: 4500 V, 200 mW, and 50 μA; starting with a voltage of 200–1500 V; the termination voltage 5000–8000 V; and a constant voltage for the protein separation zones after the application of 20 kVh.

Once the proteins were separated according to their isoelectric point, the fractions of each well were collected, and their identification was carried out. For identification, protein fractions were analyzed on a LTQ Orbitrap XL mass spectrometer equipped with a nano LC Ultimate 3000 system at the Central Research Support Service (SCAI) of the University of Cordoba. Proteins have to be digested with trypsin, previously. For this, 20 mM dithiothreitol in 25 mM ammonium bicarbonate (AB) incubated for 20 min at 55 °C was added to the sample to work under reducing conditions. The mixture was cooled to room temperature and the free thiols were alkylated by adding 40 mM iodoacetamide in 25 mM AB in the dark for 20 min. Proteolytic digestion was performed by adding 12.5 ng/μL trypsin (Promega) in 25 mM AB and incubation at 37 °C overnight. The tryptic digestion was stopped by the addition of trifluoroacetic acid at a final concentration of 1% and the digested samples were finally dried in Speedvac. Then, the sample was introduced into the LTQ Orbitrap XL mass spectrometer equipped with a nano LC Ultimate 3000 system for analysis. The electrospray voltage was set at 1300 V and the capillary voltage at 50 V at 190 °C. The LTQ Orbitrap was operated in parallel mode, which allowed an accurate measurement of the precursor ion (400–1500 m/z) in the Orbitrap selection and provided 60,000 full widths at a maximum average resolution with the acquisition of three CIDs dependent on the MS/MS scanned data in the LIT for the peptide sequence, followed by three scanned HCD MS/MS data (100-2000 m/z) with resolution 7500 FWHM m/z 400 for peptide sequencing and quantification. Conditions are described more in detail in an article published previously by Moreno-García et al. (2015) [41]. For the identification of proteins, a search was made in the Proteoma Discoverer V database. 1.0 (Thermo Fisher Scientific Software, San Jose, CA, USA) against the Uniprot database and the fixed modification of carbamidomethylation in the amino acid cysteine was included and the proteome results were statistically analyzed using the same software.

Finally, the proteins identified were quantified following the Exponentially Modified Protein Abundance Index, EmPAI, a method described by Ishihama et al. (2005) [42].

### 2.4. Statistical Analysis

All tests were performed in triplicate (n = 3). The data was previously normalized with the square root and then scaled by the Pareto method, to avoid the differences introduced by the units of measurement [43].

The data was processed using the Statgraphics Centurion XVI.II statistical package, from STSC, Inc. (Rockville, MD). The methodologies applied were ANOVA and Fisher’s test for the establishment of homogeneous groups (HG), whose objective is to establish differences between the samples, with a level of significance p ≤ 0.05. In addition, a principal component analysis (PCA) was performed to find those variables that establish differences between the two types of strains studied.

In addition, a heat map was made for a quick visualization of the changes in concentration of the analyzed compounds and a correlation analysis to establish significant relationships between metabolites and proteins. Both analyses were performed with the help of the Metaboanalyst database (https://www.metaboanalyst.ca/). The correlation matrix shows the relationships between all the variables and compares them one by one, which results in a set of data whose Pearson correlation coefficient (r) is in a range of −1 to 1. A value of correlation coefficient closes to −1 indicates that there is a strong and inverse linear relationship between the variables, while a correlation coefficient value close to 1 indicates a strong and direct linear relationship between the variables. Correlation coefficient values close to 0 are non-significant values and, therefore, it is assumed that there is no linear relationship between the variables.

## 3. Results and Discussion

A total of 20 esters were identified; 15 were identified in both strains, however, the ethyl hexanoate and ethyl lactate were only identified in the flor yeast while isobutyl acetate, ethyl 2-methyl butanoate, ethyl 3 -methyl butanoate in the P29 strain (Table 2 and Supplementary Materials). The five proteins involved in the ester metabolism were identified in this study at low concentrations [8,30,44,45,46,47,48,49]. These proteins were Atf1p, Atf2p, Iah1p, Eeb1p, and Eht1p. Besides, Atf2p was not found in the P29 strain and Eeb1p in the G1 strain. Iah1p was the most abundant protein and it was found in most conditions (Table 3 and Supplementary Materials).

The esters were classified into 6 acetate esters (ethyl acetate, methyl acetate, isobutyl acetate, isoamyl acetate, hexyl acetate, and phenylethyl acetate) and 14 ethyl esters (ethyl lactate, ethyl propanoate, ethyl isobutanoate, ethyl butanoate, ethyl 2-methyl butanoate, ethyl 3-methyl butanoate, ethyl hexanoate, ethyl heptanoate, ethyl octanoate, ethyl 2-methyl octanoate, ethyl decanoate, ethyl dodecanoate, ethyl tetradecanoate, and ethyl hexadecanoate).

**Figure 1:**
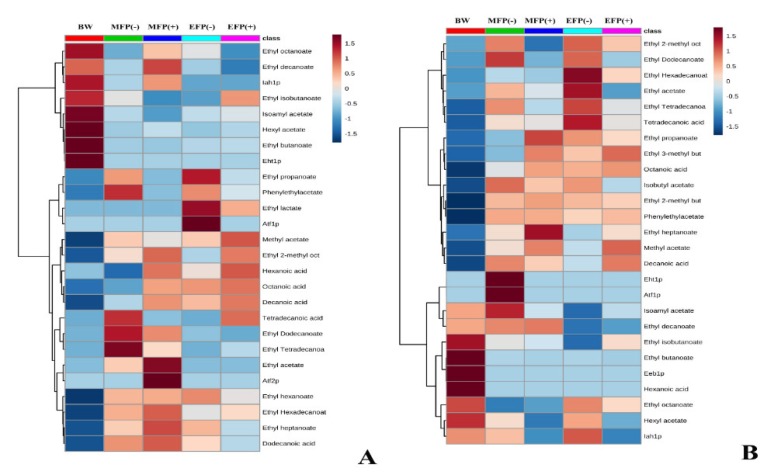
Heat map of the group of metabolites and proteins for flor yeast (**A**) and the P29 strain (**B**). *The dendrograms that indicate the grouping of variables and can be visualized in the left of the diagram. The key shown indicates the tones associated with values in the cells. In order to make full use of the shadow information, the data are normalized. The average of each condition is represented in the first top row: base wine, BW; middle of the fermentation without CO_2_ overpressure, MFP (−), middle of the fermentation with CO_2_ overpressure, MFP (+); final fermentation without CO_2_ overpressure, EFP (−); final fermentation with CO_2_ overpressure, EFP (+).

The evolution of the metabolites as well as of the proteins is represented throughout the second fermentation process for both pressure and without CO_2_ pressure conditions for both strains by a heatmap (Figure 1). In this way, a general and integrated view of the metabolome and proteome involved in this process is obtained, allowing a first approach. Figure 1A showed how hexanoic acid or dodecanoic acid, as well as ethyl hexanoate, were present in higher concentrations in the G1 strain. The enzyme Atf2p also stands out, whose concentration increased in the middle of the second fermentation under pressure conditions. Figure 1B exhibited that esters such as phenylethyl acetate, ethyl 3-methylbutanoate, isobutyl acetate, ethyl 2-methylbutanoate, or acids such as octanoic acid and decanoic acid were found at a higher concentration throughout the fermentation process both understudy conditions and in the P29 strain. As for the proteins, the proteins Eht1p and Atf1p showed a high concentration in the P29 strain in the middle of the second fermentation in the condition without CO_2_ pressure, suggesting a possible relationship between them. Eht1p was also important in the P29 strain in the no-pressure condition, although the metabolites associated with this strain did not reduce its concentrations as would be expected since there is a degradation enzyme. Finally, the Eeb1p enzyme showed a high content in the base wine of the P29 strain. A joint analysis of the heatmap data for both G1 and P29 strains showed that compounds such as ethyl tetradecanoate, tetradecanoic acid, 2-methyl ethyl octanoate, or ethyl hexadecanoate showed considerable concentrations, regardless of the strain. Other metabolites such as ethyl butanoate or ethyl isobutanoate are associated with the base wine, their concentration is reduced during the fermentation process in both strains and conditions.

### 3.1. Acetate Esters

In the condition without CO_2_ overpressure, the metabolomic profile obtained for the acetate esters was not similar for both strains. However, there was in both cases, an increase in the concentration of methyl acetate and a decrease in the concentrations of hexyl acetate, and isoamyl acetate was obtained (Table 2 and Supplementary Materials). According to the literature, compounds of hexyl acetate and phenylethyl acetate decrease during cava production, so that they could be used as aging markers [50].

In the P29 strain, the amount of isobutyl acetate increased (0.01 mg/L), not being identified in flor yeast (G1 strain). This concentration is in agreement with the range established for the isobutyl acetate, between 0.01–0.8 mg/L in wine [51]. Ethyl acetate, the main ester produced in wine [51], was produced in high amounts in both strains (20–22 mg/L). Isoamyl acetate (2–5 mg/L) and 2–phenylethyl acetate (0.40–0.86 mg/L) constituted the following important ester compounds in the sparkling wines analyzed [28].

**Figure 2:**
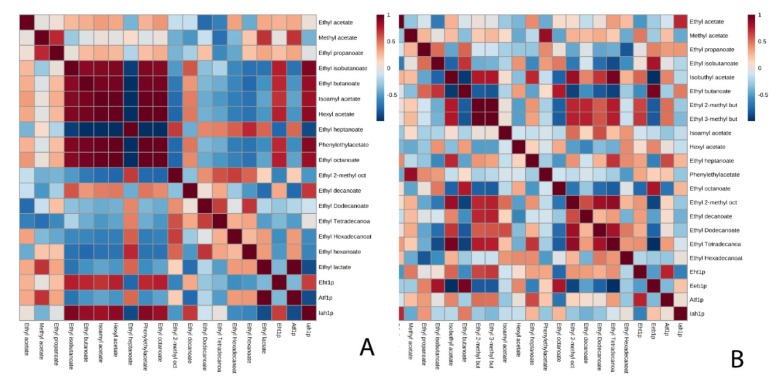
Matrix resulting from the analysis of correlations made for the condition without pressure in flor yeast (**A**) and in the P29 strain (**B**) with a level of significance of 95%.

With respect to the proteins involved in the synthesis and degradation of these compounds, the behavior was different in each strain (Table 3 and Supplementary Materials). The protein that presented the highest protein content was Iah1p in both strains. In flor yeast, both proteins (Atf1p and Iah1p) were identified, Atf1p being the one that presented higher protein content at the end of the second fermentation (0.032 mol%). The correlation analysis (Figure 2A) showed a measured correlation with methyl acetate (0.692). During the course of the fermentation, the content of this protein and the concentration of this compound increased. Conversely, the behavior obtained for the proteins that intervened in the metabolism of the esters of the control strain (P29 strain) differed from the flor strain. In the P29 strain, Atf1p was identified in MF and not in the other conditions; and the protein content of Iah1p decreased in MF and increased in EF. In addition, in the correlation analysis shown in Figure 2B for Atf1p, it was observed that there was no significant correlation and the correlations obtained for Iah1p were close to zero, and no significant correlation could be established.

It would be interesting to highlight the correlation obtained between an acetate ester synthesis protein with an ethyl ester in flor yeast. The correlation analysis (Figure 2A) presented a strong correlation between Atf1p and ethyl lactate (0.999). During the course of the fermentation, the content of this protein and the concentration of this compound increased. Ethyl lactate is predominantly produced during alcoholic fermentation from reactions between alcohols and Acetyl-CoA catalyzed by alcohol acetyltransferase [17]. In this study, ethyl lactate was identified at the end of the second fermentation in both conditions as in Ubeda et al. (2019) [28]. In addition, ethyl lactate has also been previously reported as a good marker of aging [52]. This increase is very interesting and it differentiates the behavior of this yeast compared to the P29 strain. On the other hand, a strong and inverse correlation was observed between Iah1p and ethyl lactate (–0.867), that is, as the protein content of Iah1p decreases, the amount of ethyl lactate increases.

Meanwhile, in the CO_2_ overpressure condition, the metabolic behavior of both strains was different. In the P29 strain, there was an increase in the concentration of methyl acetate and isobutyl acetate in MF, remained unchanged at the end, EF. In the flor yeast, a significant decrease in the amount of most acetate esters was observed during the second fermentation, except for methyl acetate, whose concentration increased, and isobutyl acetate that was not identified. The concentration of the rest of acetate esters remained without significant differences during this study; except hexyl acetate that decreased as in the studies carried out by other authors [53].

**Figure 3:**
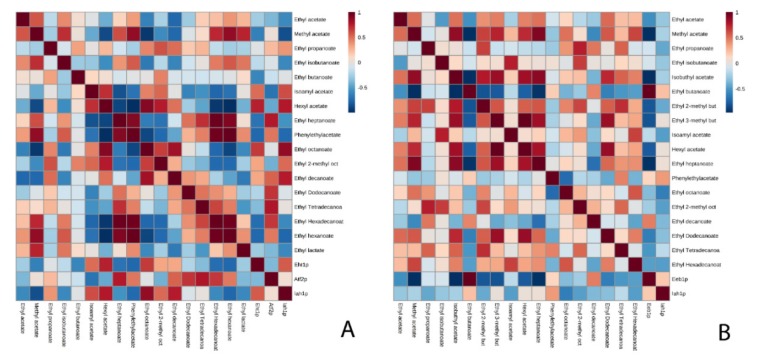
Matrix resulting from the analysis of correlations made for the CO_2_ overpressure condition in flor yeast (**A**) and in the P29 strain (**B**) with a confidence level of 95%.

The difference between both strains for the proteins involved in the formation and degradation of acetate esters (Atf1p, Atf2p, and Iah1p) was the detection of Atf2p in MF in the flor yeast, whereas in the P29 strain, this protein was not detected. Iah1p protein, showed an inverse correlation with phenylethyl acetate (–0.749) in flor yeast (Figure 3A); while in the P29 strain (Figure 3B), there was no correlation between Iah1p and phenylethyl acetate.

In this study, the behavior of each strain in terms of CO_2_ overpressure was not affected, except for hexyl acetate and phenylethyl acetate, whose amount was greater in the CO_2_ overpressure condition. However, with regard to the proteins, greater differences were found in terms of CO_2_ overpressure, since Atf1p was not identified in both strains (Table 3 and Supplementary Materials). It seems that the synthesis of this protein could be repressed due to the overpressure to which yeasts are subjected because it has only been identified in no-pressure condition. However, more experiments are needed to corroborate this hypothesis.

It can be concluded after this study that the metabolic profile obtained is practically identical both with pressure and without pressure for each strain. This implies that the overpressure of CO_2_ is not involved in the metabolism of acetate esters.

### 3.2. Ethyl Esters

With regards to the production of ethyl esters in sparkling wines, results depend on the yeast strain, the growth conditions, and the stress of the yeast [54]. Changes can be explained by the mechanism of adsorption–desorption of the cell walls and by the enzymatic or chemical hydrolysis of its esters, related to the phenomenon of autolysis of the yeast [32].

In the condition without CO_2_ overpressure, the ethyl ester that showed the highest concentration in both strains was ethyl octanoate (1.5–3 mg/L). This compound confers sour and apple aroma [50]. This result was also reported by several authors such as López de Lerma et al. (2018) [55] in cava wines that use yeast strains in free forms, bio-immobilized or immobilized with alginate. In particular, the main compounds detected were ethyl butanoate, ethyl hexanoate, and ethyl octanoate [55]. In our study, a different behavior was obtained in both strains with respect to the metabolism of the ethyl esters, except ethyl isobutanoate, ethyl heptanoate, ethyl 2-methyl butanoate, and ethyl octanoate.

No ethyl 2-methyl butanoate and 3-methyl butanoate ethyl were identified in flor yeast (G1 strain). The concentration of 6 ethyl esters decreased during the second fermentation. In addition, the concentration of the rest of esters increased significantly. On the other hand, in the P29 strain, ethyl hexanoate and ethyl lactate were not found. Additionally, most of the esters identified showed a significant increase in their concentration during the course of the second fermentation, except for ethyl isobutanoate, ethyl dodecanoate, ethyl butanoate, and ethyl octanoate, whose concentration decreased in EF. The decrease in esters during wine aging is generally attributed to the release of hydrolytic enzymes [56], so these results would be explained.

The proteins that intervene in the metabolism of ethyl esters are Eeb1p, Eht1p, and Iah1p. In flor yeast, Eht1p was identified in the base wine and Iah1p decreased its protein content during the second fermentation and it was not identified in EF. In contrast, in the P29 strain, Eht1p was identified only in MF, Eeb1p in the base wine, and the content of Iah1p decreased in MF but increased in EF. Such as the study of acetate esters, a correlation analysis was performed in order to identify correlations between proteins and ethyl esters. It was possible to establish in this case a greater number of correlations in the flor yeast (Figure 2A); for Eht1p, the strongest and most direct correlations were with ethyl isobutanoate (0.755), ethyl butanoate (0.730), and ethyl octanoate (0.756). If the protein content of Eht1p increases, the amount of these ethyl esters also increases. The concentration of ethyl heptanoate increases as the content of Iah1p decreases (–0.877), so in this case a strong and inverse correlation was established. The correlations established in the strain P29 for Eeb1p were the same as those of the flor yeast for Eht1p; ethyl isobutanoate (0.801), ethyl butanoate (0.960), and ethyl octanoate (0.796); while those established for Eht1p were with ethyl 2-methyl butanoate (0.634) and ethyl 3-methyl butanoate (0.678). However, no correlation of interest could be established between Iah1p and the rest of the ethyl esters (Figure 2B).

With regard to the CO_2_ overpressure condition, in flor yeast, the amount of ethyl dodecanoate, ethyl hexadecanoate, ethyl heptanoate, ethyl propanoate, and ethyl decanoate was affected in EF, whereas in the P29 strain only its concentration of ethyl dodecanoate and ethyl heptanoate were affected. The CO_2_ overpressure could be affecting the amount of ethyl heptanoate in EF, causing a decrease. The major difference observed in the flor yeast in the CO_2_ overpressure condition was with ethyl propanoate, since this compound increased its content in EF while in the non-pressure CO_2_ condition, it declined. The same happened in the P29 strain for ethyl dodecanoate: without pressure, this compound decreased its concentration in EF, while with CO_2_ overpressure its amount increased during the second fermentation.

Regarding the protein analysis, in the flor yeast, a smaller number of proteins was identified than in P29. No protein was identified in EF in both strains. Eht1p and Iah1p were the ones with the highest protein content in P29. The number of correlations established between the proteins and the ethyl esters for the yeast was less than in the previous condition. In flor yeast, Eht1p could be correlated with ethyl octanoate (0.668) and Iah1p with ethyl hexanoate (–0.765) (Figure 3A). In the P29 strain, it could only be related to Eeb1p with ethyl butanoate (0.999) (Figure 3B).

**Figure 4:**
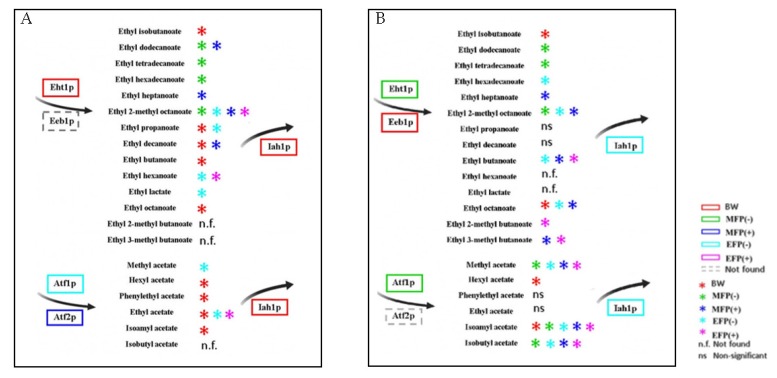
Summary scheme of metabolites and proteins that presented a higher concentration in each study concentration and are involved with the metabolism of esters for flor yeast (**A**) and the strain P29 (**B**). The color of the asterisks and boxes represent the condition in which the metabolites and proteins were identified, respectively. Each condition is represented by a color: red for the base wine, BW; green for the middle of the fermentation without CO_2_ overpressure, MFP (−); dark blue for half fermentation with CO_2_ overpressure, MFP (+); light blue final fermentation without CO_2_ overpressure, EFP (−); pink for final fermentation with CO_2_ overpressure, EFP (+).

The metabolites and proteins that presented the highest concentration in each strain are highlighted in a schematic figure (Figure 4) in order to provide a better understanding of the results obtained in this work.

**Figure 5:**
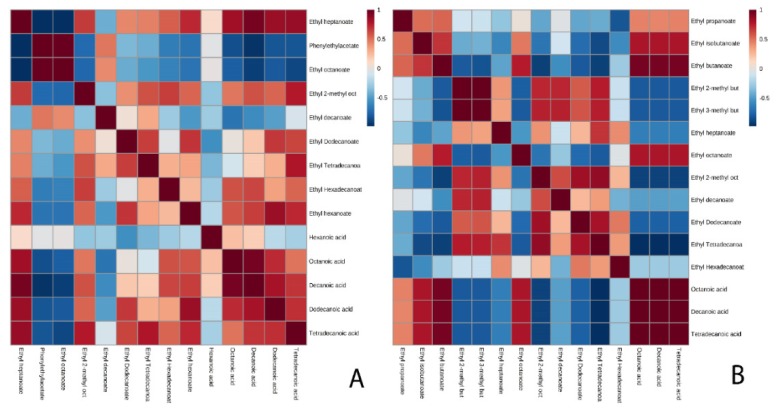
Matrix of correlations established between esters and fatty acids in the condition without CO_2_ overpressure for flor yeast (**A**) and the P29 strain (**B**).

**Figure 6:**
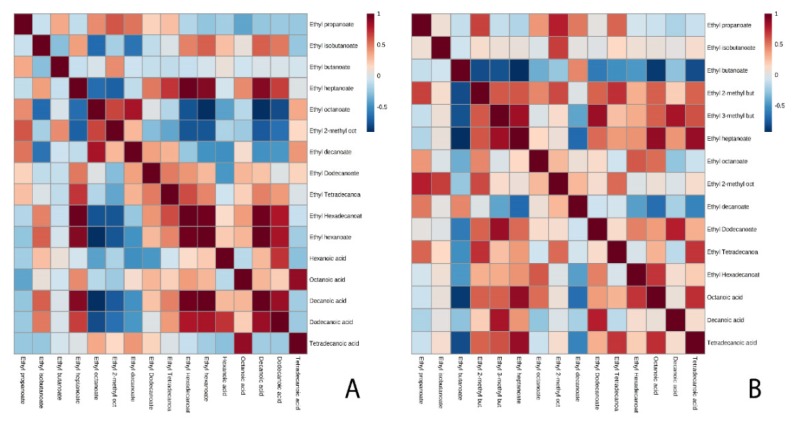
Matrix of correlations established between esters and fatty acids in the CO_2_ overpressure condition for the flor yeast (**A**) and the P29 strain (**B**).

In order to perform a deeper study of the metabolism of ethyl esters, it was decided to study the potential substrates, that are, the medium chain fatty acids (Table 4 and Supplementary Materials). Five fatty acids (hexanoic acid, octanoic acid, decanoic acid, dodecanoic acid, and tetradecanoic acid) were identified in flor yeast, while 3 acids (octanoic acid, decanoic acid, and tetradecanoic acid) were identified in the P29 strain. In addition, correlations were established between ethyl esters and fatty acids, highlighting a greater number of correlations in the flor yeast compared to the P29 strain. In both conditions for the flor yeast, the fatty acids were related to ethyl heptanoate and ethyl hexanoate (Figure 5A and Figure 6A). On the contrary, in the P29 strain, the correlations that were established in both conditions were different (Figure 5B and Figure 6B), highlighting in the CO_2_ overpressure condition, the correlation with ethyl heptanoate and the condition without pressure, the relationship with ethyl butanoate.

**Figure 7:**
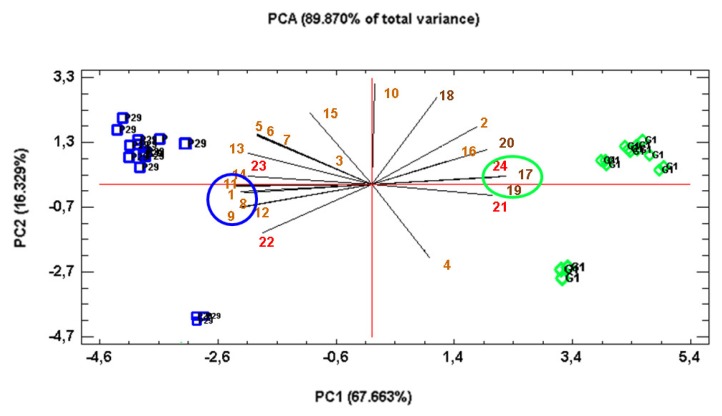
Principal component analysis, PCA. *The different compounds were identified by numbers as follows: 1. Ethyl acetate, 2. Methyl acetate, 3. Ethyl propanoate, 4. Ethyl isobutanoate, 5. Isobutyl acetate, 6. Ethyl 2-methyl butanoate, 7. Ethyl 3-methyl butanoate, 8. Isoamyl acetate, 9. Hexyl acetate, 10. Ethyl heptanoate, 11. Phenylethyl acetate, 12. Ethyl octanoate, 13. Ethyl 2-methyl octanoate, 14. Ethyl decanoate, 15. Ethyl tetradecanoate, 16. Ethyl lactate, 17. Hexanoic acid, 18. Octanoic acid, 19. Tetradecanoic acid, 20. Dodecanoic acid, 21. Eht1p, 22. Eeb1p, 23. Atf1p, 24. Atf2p. The blue color for the P29 strain and green for the G1 strain (flor yeast) were used. Proteins (21–24) were represented in red, fatty acid esters (1–16) in dark yellow and fatty acids (17–20) in dark brown.

Finally, a multivariate analysis of the principal components was carried out to discriminate between the variables those that separate the flor yeast from the P29 strain. The PCA obtained (Figure 7) explained 89.870% of the total variance. PC1 (67.663%) separated with positive values the flor yeast to the P29 strain, with negative values. The variables that separated the flor yeast were hexanoic acid (0.246), tetradecanoic acid (0.246), and Atf2p (0.234). However, the acetate esters were the variables that discriminated against the P29 strain. These esters were ethyl acetate (–0.246), isoamyl acetate (–0.246), hexyl acetate (–0.241), and phenylethyl acetate (–0.247).

## 4. Conclusions

This work focuses on investigating the possible use of a flor yeast for the production of sparkling wines, through the study of the metabolism of esters, and due to its strong influence on the organoleptic properties of sparkling wines. In general, an insignificant effect of CO_2_ overpressure on quantified esters was observed since the two strains analyzed showed a similar behavior during the second fermentation under both study conditions. Furthermore, in flor yeast, the content of proteins responsible for the synthesis and degradation of esters did not change versus the P29 strain, giving the possibility of establishing a greater number of significant correlations between esters and proteins in a flor yeast.

With regard to the compounds produced by the flor yeast that are of great organoleptic interest for sparkling wines, in addition to exhibiting fermentation characteristics similar to those obtained by conventional yeast, it can be concluded that this is a yeast strain suitable for the production of this special type of wine, thus increasing yeast strain biodiversity and possibly reducing production costs since this wine strain facilitates the clarification and subsequent removal of the wine, thanks to its capacity of cell–cell adhesion. These results suppose a first approximation in the search of metabolome–proteome relations of the yeast during the elaboration of the sparkling wine (cava). However, further research that considers the study of other metabolic compounds and pathways, as well as a transcriptomic and genetic approach and enzymatic activities, would be necessary in order to achieve more solid conclusions.

## Figures and Tables

**Table 1:** Amount of viable cells in the studied conditions (CFU/mL).

	BW	MFP (−)	EFP (−)	MFP (+)	EFP (+)
**G1 strain**	1.5 × 10^6^ ± 0	3.71 × 10^6^ ± 2.3x10^5^	7.12 × 10^5^ ± 1.2x10^5^	2.87 × 10^6^ ± 4.9x10^5^	3.5 × 10^3^ ± 7.07x10^2^
**P29 strain**	1.5 × 10^6^ ± 0	5.53 × 10^6^ ± 2.1x10^6^	1.14 × 10^6^ ± 4.2x10^5^	8.02 × 10^6^ ± 1.4x10^6^	3.33 × 10^4^ ± 1.2x10^4^

* BW: base wine; MFP (+): middle of fermentation with pressure; MFP (−): middle of fermentation without pressure; EFP (+): end of fermentation with pressure; EFP (−): end of fermentation without pressure.

**Table 2:** Determination of the concentration (mg/L) of esters by gas chromatograph coupled to flame ionization detector (GC-FID) and stir-bar sorptive extraction, followed by thermal desorption and gas chromatography-mass spectrometry (SBSE-TD-GC-MS).

	G1 Strain					P29 Strain				
	Concentration (mg/L)									
	BW	MFP (−)	EFP (−)	MFP (+)	EFP (+)	BW	MFP (−)	EFP (−)	MFP (+)	EFP (+)
**Methyl acetate**	0.071^a^±0.008	0.132^b^±0.004	0.257^d^±0.006	0.140^b^±0.001	0.18^c^±0.03	0.071^a^±0.008	0.25^b^±0.09	0.22^b^±0.03	0.32^b^±0.05	0.30^b^±0.03
**Hexyl acetate**	0.256^d^±0.007	0.124^ab^±0.003	0.102^a^±0.004	0.144^c^±0.003	0.122^bc^±0.002	0.256^c^±0.007	0.23^ab^±0.02	0.23^b^±0.01	0.20^a^±0.04	0.22^a^±0.02
**Phenylethyl acetate**	0.76^c^±0.02	0.41^a^±0.03	0.411^a^±0.009	0.51^b^±0.04	0.49^b^±0.03	0.76^ns^±0.02	0.84^ns^±0.06	0.82^ns^±0.05	0.79^ns^±0.08	0.81^ns^±0.07
**Ethyl acetate**	19.9^b^±0.8	12^a^±3	20.2^b^±0.9	13.9^a^±0.4	14^b^±4	19.9^ns^±0.8	21^ns^±3	21.69^ns^±0.02	21.4^ns^±0.7	22^ns^±5
**Isoamyl acetate**	6.1^c^±0.7	2.50^a^±0.01	2.4a^b^±0.4	2.23^a^±0.02	2.2^b^±0.8	6.1^b^±0.7	5.8^b^±0.4	4.7^b^±0.4	5.1^ab^±0.3	5.1^ab^±0.5
**Isobutyl acetate**	n.f.	n.f.	n.f.	n.f.	n.f.	n.f.^a^	0.010^b^±0.002	0.011^b^±0.001	0.009^b^±0.001	0.009^b^±0.002
**Ethyl isobutanoate**	0.0032^d^±0.0004	0.0013^b^±0.0001	0.0010^a^±0.0004	0.0009^a^±0.0002	0.0026^c^±0.0001	0.0032^c^±0.0004	0.0015^b^±0.0002	0.0004^a^±0.0005	0.0014^b^±0.0001	0.0016^b^±0.0005
**Ethyl dodecanoate**	0.0005^a^±0.0001	0.0183^c^±0.0008	0.0017^b^±0.0003	0.021^c^±0.001	0.0004^a^±0.0002	0.0005^a^±0.0001	0.053^e^±0.002	0.031^d^±0.003	0.0061^b^±0.0006	0.008^c^±0.001
**Ethyl tetradecanoate**	n.f.^a^	0.00174^d^±0.0003	n.f.^a^	0.0003^c^±0.0006	0.0002^b^±0.0009	n.f.^a^	0.0012^cd^±0.0001	0.0013^d^±0.0001	0.0007^b^±0.0001	0.0012^b^c±0.0001
**Ethyl hexadecanoate**	0.0021^a^±0.0003	0.0077^e^±0.0002	0.0051^b^±0.0002	0.0065^d^±0.0002	0.0059^c^±0.0002	0.0021^a^±0.0003	0.0027^b^±0.0001	0.007^c^±0.004	0.0030^b^±0.0006	0.0035^b^±0.0006
**Ethyl heptanoate**	n.f.^a^	0.006^c^±0.001	0.006^c^±0.002	0.007^d^±0.001	0.002^b^±0.001	n.f.^a^	0.009^b^±0.002	0.008^b^±0.003	0.028^c^±0.003	0.0094^b^±0.00004
**Ethyl 2-methyl octanoate**	0.0031^a^±0.0002	0.0041^b^±0.0003	0.0035^ab^±0.0003	0.0048^b^±0.0004	0.0048^b^±0.0006	0.0031^a^±0.0002	0.0044^b^±0.0002	0.0045^ab^±0.0001	0.0046^b^±0.0003	0.00370^a^±0.00002
**Ethyl propanoate**	0.187^c^±0.005	0.129^b^±0.003	0.173^c^±0.004	0.0687^a^±0.0002	0.130^b^±0.001	0.187^ns^±0.005	0.17^ns^±0.03	0.19^ns^±0.01	0.21^ns^±0.06	0.20^ns^±0.01
**Ethyl decanoate**	0.66^d^±0.01	0.56^c^±0.03	0.35^b^±0.02	0.67^d^±0.02	0.23^a^±0.01	0.66^ns^±0.01	0.65^ns^±0.02	0.64^ns^±0.03	0.69^ns^±0.01	0.5^ns^±0.2
**Ethyl butanoate**	2.18^d^±0.01	0.201^a^±0.002	0.32^b^±0.01	0.202^a^±0.002	0.41^c^±0.01	2.18^a^±0.01	0.26^b^±0.01	0.25^bc^±0.02	0.30^c^±0.01	0.29^bc^±0.01
**Ethyl hexanoate**	n.f.^a^	0.771^b^±0.002	0.86^c^±0.03	0.78^b^±0.01	0.85^c^±0.03	n.f.	n.f.	n.f.	n.f.	n.f.
**Ethyl lactate**	n.f.^a^	n.f.^a^	45^c^±2	n.f.^a^	31^b^±5	n.f.	n.f.	n.f.	n.f.	n.f.
**Ethyl octanoate**	2.799^d^±0.001	1.621^b^±0.002	1.5^b^±0.01	1.99^c^±0.03	1.26^a^±0.04	2.799^b^±0.001	2.2^a^±0.2	2.45^ab^±0.03	2.6^b^±0.2	2.1^a^±0.1
**Ethyl 2-methyl butanoate**	n.f	n.f.	n.f.	n.f.	n.f.	n.f.^a^	0.0161^b^±0.0001	0.0158^b^±0.0001	0.0163^b^±0.001	0.0221^c^±0.0002
**Ethyl 3-methyl butanoate**	n.f.	n.f.	n.f.	n.f.	n.f.	n.f.^a^	0.028^b^±0.001	0.026^b^±0.002	0.034^c^±0.001	0.036^c^±0.002

* Different letters (a-b) in the same row are significantly different (*p* ≤ 0.05) according to Fisher’s test, ns = non-significant. BW: base wine; MFP (+): middle of fermentation with pressure; MFP (−): middle of fermentation without pressure; EFP (+): end of fermentation with pressure; EFP (−): end of fermentation without pressure. ** n.f.—not found

**Table 3:** Determination of protein content (mol%) by the abundance index (EmPAI) of the proteins identified in each condition related to the esters metabolism.

	G1 Strain					P29 Strain				
	Protein content (mol%)									
	BW	MFP (−)	EFP (−)	MFP (+)	EFP (+)	BW	MFP (−)	EFP (−)	MFP (+)	EFP (+)
Atf1p	n.f.^a^	n.f.^a^	0.032^b^±0.005	n.f.^a^	n.f.^a^	n.f.^a^	0.004^b^±0.002	n.f.^a^	n.f.^a^	n.f.^a^
Atf2p	n.f.^a^	n.f.^a^	n.f.^a^	0.011^b^±0.003	n.f.^a^	n.f.	n.f.	n.f.	n.f.	n.f.
Eht1p	0.007^b^±0.001	n.f.^a^	n.f.^a^	n.f.^a^	n.f.^a^	n.f.^a^	0.022^b^±0.001	n.f.^a^	n.f.^a^	n.f.^a^
Eeb1p	n.f.	n.f.	n.f.	n.f.	n.f.	0.012^b^±0.004	n.f.^a^	n.f.^a^	n.f.^a^	n.f.^a^
Iah1p	0.033^d^±0.003	0.009^b^±0.001	n.f.^a^	0.022^c^±0.003	n.f.^a^	0.021^d^±0.001	0.018^b^±0.001	0.025^e^±0.002	0.0195^c^±0.0002	n.f.^a^

* Different letters (a-b) in the same row are significantly different (*p* ≤ 0.05) according to Fisher’s test. BW: base wine; MFP (+): middle of fermentation with pressure; MFP (−): middle of fermentation without pressure; EFP (+): end of fermentation with pressure; EFP (−): end of fermentation without pressure. ** n.f.—not found

**Table 4:** Determination of the concentration (mg/L) of fatty acids involved in the synthesis of ethyl esters.

	G1 Strain					P29 Strain				
	Concentration (mg/L)									
	BW	MFP (−)	EFP (−)	MFP (+)	EFP (+)	BW	MFP (−)	EFP (−)	MFP (+)	EFP (+)
Hexanoic acid	0.137^a^±0.005	0.203^b^±0.006	0.20^b^±0.02	0.26^c^±0.03	0.26^c^±0.01	0.137^a^±0.005	n.f.	n.f.	n.f.	n.f.
Octanoic acid	12.67^a^±0.06	18^b^±2	28^cd^±1	26^c^±1	28.4^d^±0.2	12.67^a^±0.06	31^b^±2	32^b^±1	31^b^±3	35^b^±2
Decanoic acid	0.91^a^±0.03	2.02^b^±0.01	2.7^c^±0.3	3.2^d^±0.1	3.22^d^±0.06	0.91^a^±0.03	3.2^b^±0.4	3.3^b^±0.4	3.0^b^±0.1	3.6^b^±0.5
Tetradecanoic acid	0.814^a^±0.002	0.93^c^±0.01	0.86^b^±0.01	0.871^b^±0.006	0.863^b^±0.003	0.814^a^±0.002	0.11^a^±0.03	0.17^b^±0.01	0.119^a^±0.004	0.1^a^±0.1
Dodecanoic acid	n.f.^a^	0.149^c^±0.001	0.125^b^±0.002	0.169^d^±0.006	0.119^b^±0.007	n.f.	n.f.	n.f.	n.f.	n.f.

* Different letters (a-b) in the same row are significantly different (*p* ≤ 0.05) according to Fisher’s test. BW: base wine; MFP (+): middle of fermentation with pressure; MFP (−): middle of fermentation without pressure; EFP (+): end of fermentation with pressure; EFP (−): end of fermentation without pressure. ** n.f.—not found

## Supplementary Materials

The following are available online at https://www.mdpi.com/2076-2607/8/3/403/s1.

## References

[1] Belda I., Ruiz J., Esteban-Fernández A., Navascués E., Marquina D., Santos A., Moreno-Arribas M. (2017). Microbial Contribution to Wine Aroma and its Intended use for Wine Quality Improvement. Molecules.

[2] Kurtzman C.P., Fell J.W., Boekhout T. (2011). The Yeasts: A Taxonomic Study.

[3] Rapp A., Mandery H. (1986). Wine Aroma. Experientia.

[4] Suomalainen H. (1971). Yeast and its Effect on the Flavour of Alcoholic Beverages. J. Inst. Brew..

[5] Soufleros E.H., Bertrand A. (1979). Rôle de la Souche de Levure Dans la Production des Substances Volatiles au Cours de la Fermentation du jus de Raisin. OENO One.

[6] Lee S.J., Rathbone D., Asimont S., Adden R., Ebeler S.E. (2014). Dynamic Changes in Ester Formation During Chardonnay Juice Fermentations with Different Yeast Inoculation and Initial Brix Conditions. Am. J. Enol. Vitic..

[7] Mateo J.J., Jimenez M., Huerta T., Pastor A. (1992). Comparison of Volatiles Produced by Four *Saccharomyces Cerevisiae* Strains Isolated from Monastrell Musts. Am. J. Enol. Vitic..

[8] Plata M.C., Mauricio J.C., Millán C., Ortega J.M. (1998). In Vitro Specific Activity of Alcohol Acetyltransferase and Esterase in Two Flor Yeast Strains during Biological Aging of Sherry Wines. J. Ferment. Bioeng..

[9] Plata M.C., Millán C., Mauricio J.C., Ortega J.M. (2003). Formation of Ethyl Acetate and Isoamyl Acetate by Various Species of Wine Yeasts. Food Microbiol..

[10] Stribny J., Gamero A., Pérez-Torrado R., Querol A. (2015). *Saccharomyces Kudriavzevii* and *Saccharomyces uvarum* Differ from Saccharomyces Cerevisiae during the Production of Aroma-Active Higher Alcohols and Acetate Esters Using Their Amino Acidic Precursors. Int. J. Food Microbiol..

[11] Walker G., Stewart G. (2016). *Saccharomyces Cerevisiae* in the Production of Fermented Beverages. Beverages.

[12] Di Gianvito P., Perpetuini G., Tittarelli F., Schirone M., Arfelli G., Piva A., Patrignani F., Lanciotti R., Olivastri L., Suzzi G. (2018). Impact of *Saccharomyces Cerevisiae* Strains on Traditional Sparkling Wines Production. Food Res. Int..

[13] Piendl A., Geiger E. (1980). Technological Factors in the Formation of Esters during Fermentation (Brewing technology). Brew. Dig..

[14] Rollero S., Bloem A., Camarasa C., Sanchez I., Ortiz-Julien A., Sablayrolles J.M., Dequin S., Mouret J.R. (2015). Combined Effects of Nutrients and Temperature on the Production of Fermentative Aromas by *Saccharomyces Cerevisiae* during Wine Fermentation. Appl. Microbiol. Biotechnol..

[15] Edwards C.G., Beelman R.B., Bartley C.E., McConnell A.L. (1990). Production of Decanoic Acid and other Volatile Compounds and the Growth of Yeast and Malolactic Bacteria during Vinification. Am. J. Enol. Vitic..

[16] Gómez E., Laencina J., Martinez A. (1994). Vinification Effects on Changes in Volatile Compounds of Wine. J. Food Sci..

[17] Kemp B., Alexandre H., Robillard B., Marchal R. (2015). Effect of Production Phase on Bottle-Fermented Sparkling Wine Quality. J. Agric. Food Chem..

[18] Zhang S., Petersen M., Liu J., Toldam-Andersen T. (2015). Influence of Pre-Fermentation Treatments on Wine Volatile and Sensory Profile of the New Disease Tolerant Cultivar Solaris. Molecules.

[19] Culbert J.A., McRae J.M., Condé B.C., Schmidtke L.M., Nicholson E.L., Smith P.A., Howell K.S., Boss P.K., Wilkinson K.L. (2017). Influence of Production Method on the Chemical Composition, Foaming Properties, and Quality of Australian Carbonated and Sparkling White Wines. J. Agric. Food Chem..

[20] Marais J. (2016). The effect of pH on Esters and Quality of Colombar Wine during Maturation. VITIS-J. Grapevine Res..

[21] Daudt C.E., Ough C.S. (1973). Variations in some volatile acetate esters formed during grape juice fermentation. Effects of fermentation temperature, SO_2_, yeast strain, and grape variety. Am. J. Enol. Vitic..

[22] Herraiz T., Ough C.S. (1993). Formation of Ethyl esters of Amino Acids by Yeasts during the Alcoholic Fermentation of Grape Juice. Am. J. Enol. Vitic..

[23] Martínez-Lapuente L., Apolinar-Valiente R., Guadalupe Z., Ayestarán B., Pérez-Magariño S., Williams P., Doco T. (2018). Polysaccharides, Oligosaccharides and Nitrogenous Compounds Change during the Ageing of Tempranillo and Verdejo Sparkling Wines. J. Sci. Food Agric..

[24] Plata M.C., Mauricio J.C., Millán C., Ortega J.M. (2005). Influence of Glucose and Oxygen on the Production of Ethyl Acetate and Isoamyl Acetate by a *Saccharomyces Cerevisiae* Strain during Alcoholic Fermentation. World J. Microbiol. Biotechnol..

[25] Laurent M.H., Henick-Kling T., Acree T.E. (1994). Changes in the Aroma and Odor of Chardonnay Wine Due to Malolactic Fermentation. Wein Wiss..

[26] Izquierdo-Cañas P.M., Mena-Morales A., García-Romero E. (2016). Malolactic Fermentation before or during Wine Aging in Barrels. LWT Food Sci. Technol..

[27] Nykänen L., Suomalainen H. (1983). Aroma of Beer, Wine and Distilled Alcoholic Beverages. Springer Sci. Bus. Media.

[28] Ubeda C., Kania-Zelada I., del Barrio-Galán R., Medel-Marabolí M., Gil M., Peña-Neira Á. (2019). Study of the Changes in Volatile Compounds, Aroma and Sensory Attributes during the Production Process of Sparkling Wine by Traditional Method. Food Res. Int..

[29] Verstrepen K.J., Derdelinckx G., Dufour J.P., Winderickx J., Thevelein J.M., Pretorius I.S., Delvaux F.R. (2003). Flavor-Active Esters: Adding Fruitiness to Beer. J. Biosci. Bioeng..

[30] Sumby K.M., Grbin P.R., Jiranek V. (2010). Microbial Modulation of Aromatic Esters in Wine: Current Knowledge and Future Prospects. Food Chem..

[31] Mauricio J.C., Moreno J.J., Valero E.M., Zea L., Medina M., Ortega J.M. (1993). Ester Formation and Specific Activities of in Vitro Alcohol Acetyl Transferase and Esterase by *Saccharomyces Cerevisiae* during Grape Must Fermentation. J. Agric. Food Chem..

[32] Martínez-García R., García-Martínez T., Puig-Pujol A., Mauricio J.C., Moreno J. (2017). Changes in Sparkling Wine Aroma during the Second Fermentation Under CO_2_ Pressure in Sealed Bottle. Food Chem..

[33] Martínez-García R., Roldán-Romero Y., Moreno J., Puig-Pujol A., Mauricio J.C., García-Martínez T. (2020). Use of a Flor Yeast Strain for the Second Fermentation of Sparkling Wines: Effect of Endogenous CO_2_ Over-Pressure on the Volatilome. Food Chem..

[34] Garofalo C., Berbegal C., Grieco F., Tufariello M., Spano G., Capozzi V. (2018). Selection of Indigenous Yeast Strains for the Production of Sparkling Wines from Native Apulian Grape Varieties. Int. J. Food Microbiol..

[35] Ivit N.N., Kemp B. (2018). The Impact of Non-*Saccharomyces* Yeast on Traditional Method Sparkling Wine. Fermentation.

[36] Vigentini I., Cardenas S.B., Valdetara F., Faccincani M., Panont C.A., Picozzi C., Foschino R. (2017). Use of Native Yeast Strains for in-Bottle Fermentation to Face the Uniformity in Sparkling Wine Production. Front. Microbiol..

[37] Peinado R.A., Moreno J.A., Muñoz D., Medina M., Moreno J. (2004). Gas Chromatographic Quantification of Major Volatile Compounds and Polyols in Wine by Direct Injection. J. Agric. Food Chem..

[38] Tredoux A., Villiers A., Májek P., Lynen F., Crouch A., Sandra P. (2008). Stir Bar Sorptive Extraction Combined with GC–MS Analysis and Chemometric Methods for the Classification of South African Wines According to the Volatile Composition. J. Agric. Food Chem..

[39] Vararu F., Moreno-García J., Zamfir C.I., Cotea V.V., Moreno J. (2016). Selection of Aroma Compounds for the Differentiation of Wines Obtained by Fermenting Musts with Starter Cultures of Commercial Yeast Strains. Food Chem..

[40] Bradford M.M. (1976). A Rapid and Sensitive Method for the Quantitation of Microgram Quantities of Protein Utilizing the Principle of Protein-Dye Binding. Anal. Biochem..

[41] Moreno-García J., García-Martínez T., Moreno J., Mauricio J.C. (2015). Proteins Involved in Flor Yeast Carbon Metabolism under Biofilm Formation Conditions. Food Microbiol..

[42] Ishihama Y., Oda Y., Tabata T., Sato T., Nagasu T., Rappsilber J., Mann M. (2005). Exponentially Modified Protein Abundance Index (emPAI) for Estimation of Absolute Protein Amount in Proteomics by the Number of Sequenced Peptides Per Protein. Mol. Cell. Proteom..

[43] Seisonen S., Vene K., Koppel K. (2016). The Current Practice in the Application of Chemometrics for Correlation of Sensory and Gas Chromatography Data. Food Chem..

[44] Mason A.B., Dufour J.P. (2000). Alcohol Acetyltransferases and the Significance of Ester Synthesis in Yeast. Yeast.

[45] Lilly M., Bauer F.F., Lambrechts M.G., Swiegers J.H., Cozzolino D., Pretorius I.S. (2006). The Effect of Increased Yeast Alcohol Acetyltransferase and Esterase Activity on the Flavour Profiles of Wine and Distillates. Yeast.

[46] Saerens S.M.G., Verstrepen K.J., Van Laere S.D.M., Voet A.R.D., Van Dijck P., Delvaux F.R., Thevelein J.M. (2006). The *Saccharomyces Cerevisiae EHT1* and *EEB1* Genes Encode Novel Enzymes with Medium-Chain Fatty Acid Ethyl Ester Synthesis and Hydrolysis Capacity. J. Biol. Chem..

[47] Saerens S., Thevelein J., Delvaux F. (2008). Ethyl Ester Production during Brewery Fermentation, A Review. Cerevisia.

[48] Procopio S., Qian F., Becker T. (2011). Function and Regulation of Yeast Genes Involved in Higher Alcohol and Ester Metabolism during Beverage Fermentation. Eur. Food Res. Technol..

[49] Pires E.J., Teixeira J.A., Brányik T., Vicente A.A. (2014). Yeast: The Soul of Beer’s Aroma-A Review of Flavour-Active Esters and Higher Alcohols Produced by the Brewing Yeast. Appl. Microbiol. Biotechnol..

[50] Riu-Aumatell M., Bosch-Fusté J., López-Tamames E., Buxaderas S. (2006). Development of Volatile Compounds of Cava (Spanish Sparkling Wine) during Long Ageing Time in Contact with Lees. Food Chem..

[51] Lambrechts M.G., Pretorius I.S. (2000). Yeast and its Importance to Wine Aroma-A Review. S. Afr. J. Enol. Vitic..

[52] Francioli S., Torrens J., Riu-Aumatell M., López-Tamames E., Buxaderas S. (2003). Volatile Compounds by SPME-GC as Age Markers of Sparkling Wines. Am. J. Enol. Vitic..

[53] Ruiz-Moreno M.J., Muñoz-Redondo J.M., Cuevas F.J., Marrufo-Curtido A., León J.M., Ramírez P., Moreno-Rojas J.M. (2017). The Influence of Pre-Fermentative Maceration and Ageing Factors on Ester Profile and Marker Determination of Pedro Ximenez Sparkling Wines. Food Chem..

[54] Torrens J., Urpí P., Riu-Aumatell M., Vichi S., López-Tamames E., Buxaderas S. (2008). Different Commercial yeast Strains Affecting the Volatile and Sensory Profile of Cava Base Wine. Int. J. Food Microbiol..

[55] López de Lerma N., Peinado R.A., Puig-Pujol A., Mauricio J.C., Moreno J., García-Martínez T. (2018). Influence of Two Yeast Strains in Free, Bioimmobilized or Immobilized with Alginate Forms on the Aromatic Profile of Long Aged Sparkling Wines. Food Chem..

[56] Ancín-Azpilicueta C., González-Marco A., Jiménez-Moreno N. (2009). Evolution of Esters in Aged Chardonnay Wines Obtained with Different Vinification Methods. J. Sci. Food Agric..

